# Augmented reality-based surgical navigation of pelvic screw placement: an ex-vivo experimental feasibility study

**DOI:** 10.1186/s13037-023-00385-6

**Published:** 2024-01-16

**Authors:** Sandro-Michael Heining, Vladislav Raykov, Oliver Wolff, Hatem Alkadhi, Hans-Christoph Pape, Guido A. Wanner

**Affiliations:** 1 Department of Traumatology, University Hospital Zurich, Zurich, Switzerland; 2 Department of Orthopedics & Traumatology, Landeskrankenhaus Bludenz, Bludenz, Austria; 3 Hochschule Luzern Technik & Architektur, Luzern, Switzerland; 4 Department of Radiology, University Hospital Zurich, Zurich, Switzerland; 5 Spine Clinic & Traumatology, Private Hospital Bethanien, Swiss Medical Network, Zurich, Switzerland

**Keywords:** Augmented reality, Surgical navigation, Pelvic trauma, Head-mounted display

## Abstract

**Background:**

Minimally invasive surgical treatment of pelvic trauma requires a significant level of surgical training and technical expertise. Novel imaging and navigation technologies have always driven surgical technique, and with head-mounted displays being commercially available nowadays, the assessment of such Augmented Reality (AR) devices in a specific surgical setting is appropriate.

**Methods:**

In this ex-vivo feasibility study, an AR-based surgical navigation system was assessed in a specific clinical scenario with standard pelvic and acetabular screw pathways. The system has the following components: an optical-see-through Head Mounted Display, a specifically designed modular AR software, and surgical tool tracking using pose estimation with synthetic square markers.

**Results:**

The success rate for entry point navigation was 93.8%, the overall translational deviation of drill pathways was 3.99 ± 1.77 mm, and the overall rotational deviation of drill pathways was 4.3 ± 1.8°. There was no relevant theoretic screw perforation, as shown by 88.7% Grade 0–1 and 100% Grade 0–2 rating in our pelvic screw perforation score. Regarding screw length, 103 ± 8% of the planned pathway length could be realized successfully.

**Conclusion:**

The novel innovative system assessed in this experimental study provided proof-of-concept for the feasibility of percutaneous screw placement in the pelvis and, thus, could easily be adapted to a specific clinical scenario. The system showed comparable performance with other computer-aided solutions while providing specific advantages such as true 3D vision without intraoperative radiation; however, it needs further improvement and must still undergo regulatory body approval. Future endeavors include intraoperative registration and optimized tool tracking.

## Introduction

Treating pelvic and acetabular fractures is challenging [1, 2]. In recent years, percutaneous implant placement after closed fracture reduction has gained popularity due to the availability of advanced intraoperative imaging and navigation technologies [3, 4]. Although standard X-rays and screw positions in the pelvis have been described in detail, understanding the 3-dimensional (3D) pelvic anatomy, planning of screw pathways, and intraoperative precision is challenging even for experienced pelvic surgeons. While some screw pathways can be “guided” by the bony tables of the pelvis, others can be extremely difficult to insert – especially in patients with variant or distorted anatomy [3, 5, 6].

Intraoperative 3D navigation systems (CT- or Cone-Beam-based) may increase accuracy and facilitate percutaneous drilling of these screw pathways [7–9]. However, these systems are expensive and have several drawbacks. Such systems require additional hardware in the operation room (OR). Surgical workflow and the surgeon’s attention are often interrupted, resulting in problems in hand-eye coordination. Moreover, surgical navigation typically requires alignment in 3 different 2D planes (MPR) at the same time, which interrupts the surgeon’s line of sight [10, 11].

Augmented reality-visualization technology has recently entered operating rooms [12, 13] – offering a potentially safer option for the surgeon and OR staff due to lower exposure to ionizing radiation. Herein, so-called “in-situ-visualization,” the overlay of 3D medical imaging data on the patient’s anatomy, is an intuitive way of communicating perioperative image data, thus increasing precision and improving the outcome of the surgery [10, 11, 14].

Our laboratory study evaluated the feasibility and accuracy of 3D navigation for drilling of screw pathways in pelvic trauma using an off-the-shelf head-mounted-device (HMD, HoloLens 2), special modular software (HoloMA), and tool tracking of standard surgical instruments using ArUco markers [15].

## Methods

### Surgical planning

For four identical pelvic phantoms (Typ 4060, Synbone AG, Zizers) a CT data set (DICOM) was acquired using the standard Pelvic Trauma Protocol and 1 mm slice thickness (SOMATOM Force, Siemens Healthcare GmbH, Erlangen, Germany).

**Fig. 1:**
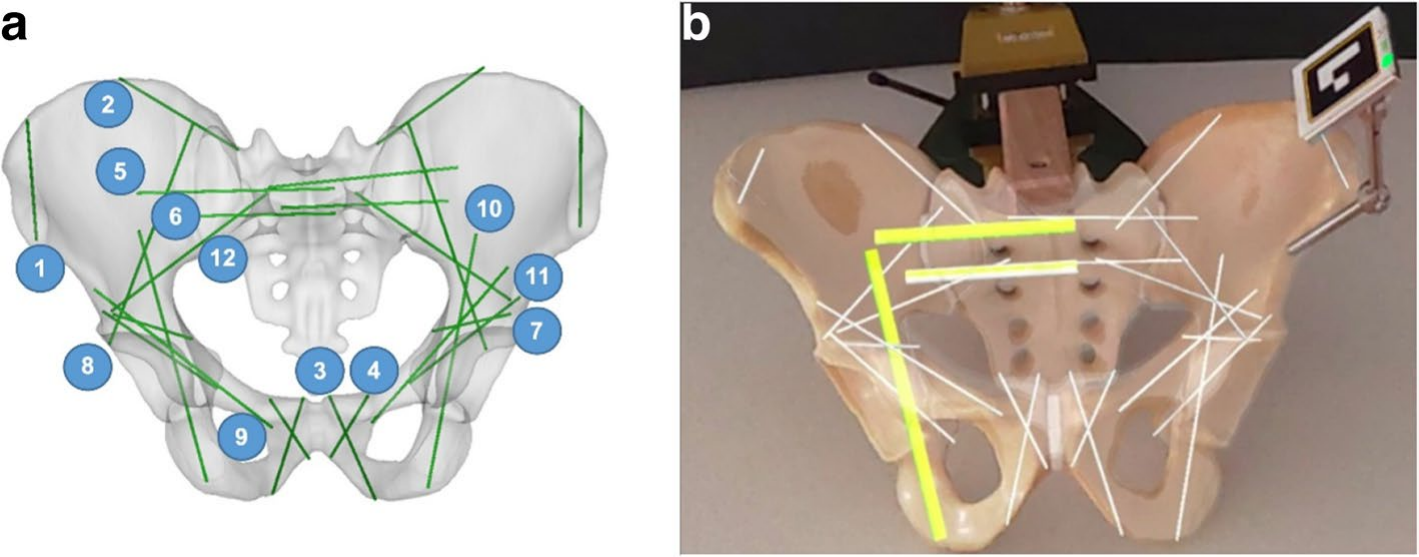
**a**) planned drill pathways, **b**) hybrid phantom

Segmentation of cortical and cancellous bone and planning of 24 standard screw positions (*n* = 12 per hemipelvis) was performed in Mimics Innovation Suite 23.0 (Materialise NV, Leuven, Belgium). The trajectories were visualized with a diameter of 1.5 mm, and positions were determined by a senior board-certified orthopedic surgeon (GAW) according to standard screw corridors (Fig. 1a).

The following trajectories representing drill pathways were planned (Table 1).

**Table 1:** Drill pathways

Screw position	Drill Pathway#
Iliac crest anterior screw	1
Iliac crest posterior screw	2
Pubic medial screw	3
Pubic lateral screw	4
Iliosacral screw S1	5
Iliosacral screw S2	6
Transverse supraacetabular screw	7
Supraacetabular screw	8
Anterior column screw	9
Posterior column screw	10
Quadrilateral plate („magic“) screw	11
S2-alar-iliac screw	12

Thereafter, 3D-image data were converted to GLB format and uploaded to HoloLens 2. After surface registration using an advanced algorithm optimized for the specific anatomical region, an overlay of the hologram, including the screw trajectories and corridors, was visualized using the HoloMA software (version 1.4 ICB-M Limited, Sofia, Bulgaria), creating hybrid phantoms (Fig. 1b).

### Trial procedure

The datasets were stored locally on three individual HoloLens 2 devices using HoloMA. The HMDs were calibrated to the three individual users and their specific vision properties.

**Fig. 2:**
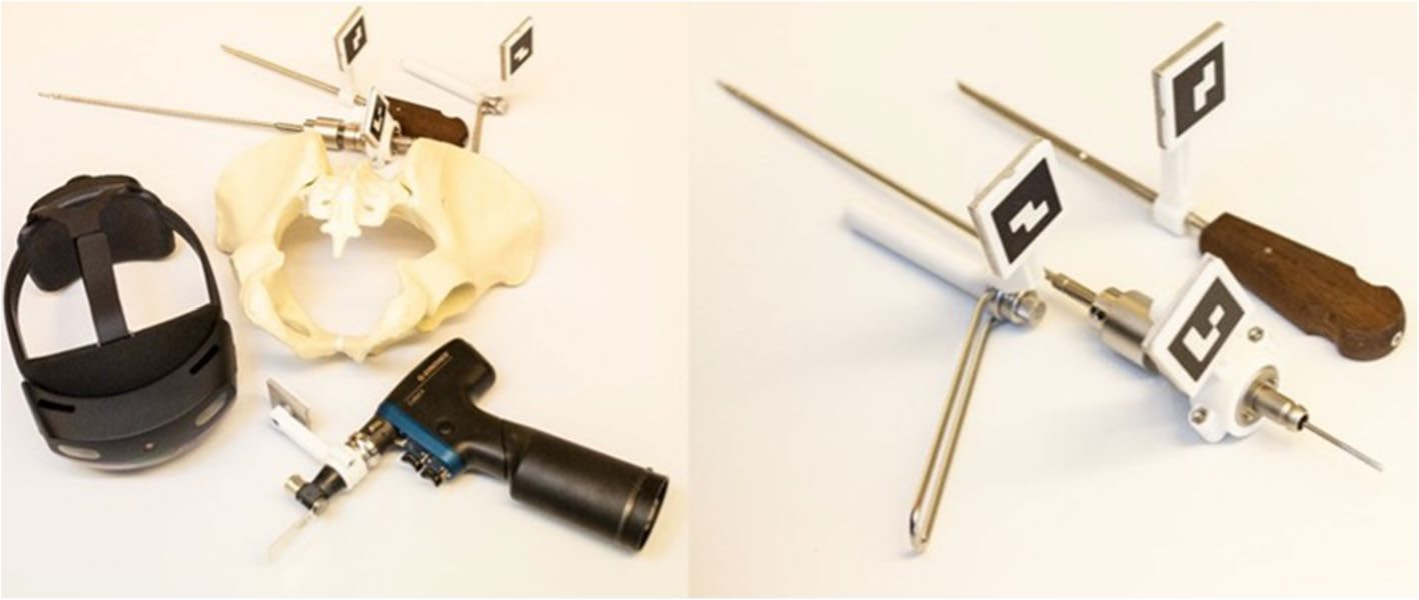
Toolset for surgical navigation in percutaneous pelvic surgery

Standard surgical instruments were augmented with 3D-printed ArUco markers, generating a trackable toolset [15]. An additional pointer was manufactured (ICB-M Limited, Sofia, Bulgaria). A pigsticker and K-wire drill sleeve adaptor for fixation of an ArUco marker for 7.3 mm cannulated screws system, the adaptors for drilling chuck, K-wire chuck, reaming chuck, and AO-rapid drilling chuck for Colibri II Powertool System (DePuy Synthes, Switzerland) were manufactured in the Additive Manufacturing Laboratory of the University of Zurich, Switzerland, using biocompatible polyamide PA2200 (Fig. 2).

**Fig. 3:**
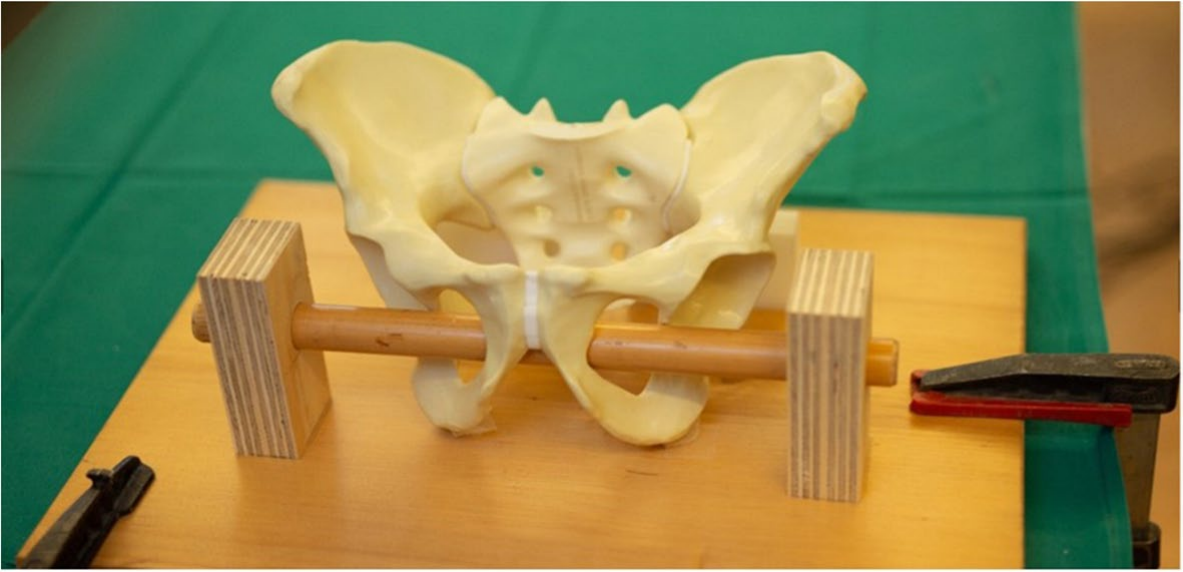
Pelvic phantom mount

The phantoms were mounted to a pelvic holder, engineered at the hospital’s workshop (Fig. 3).

**Fig. 4:**
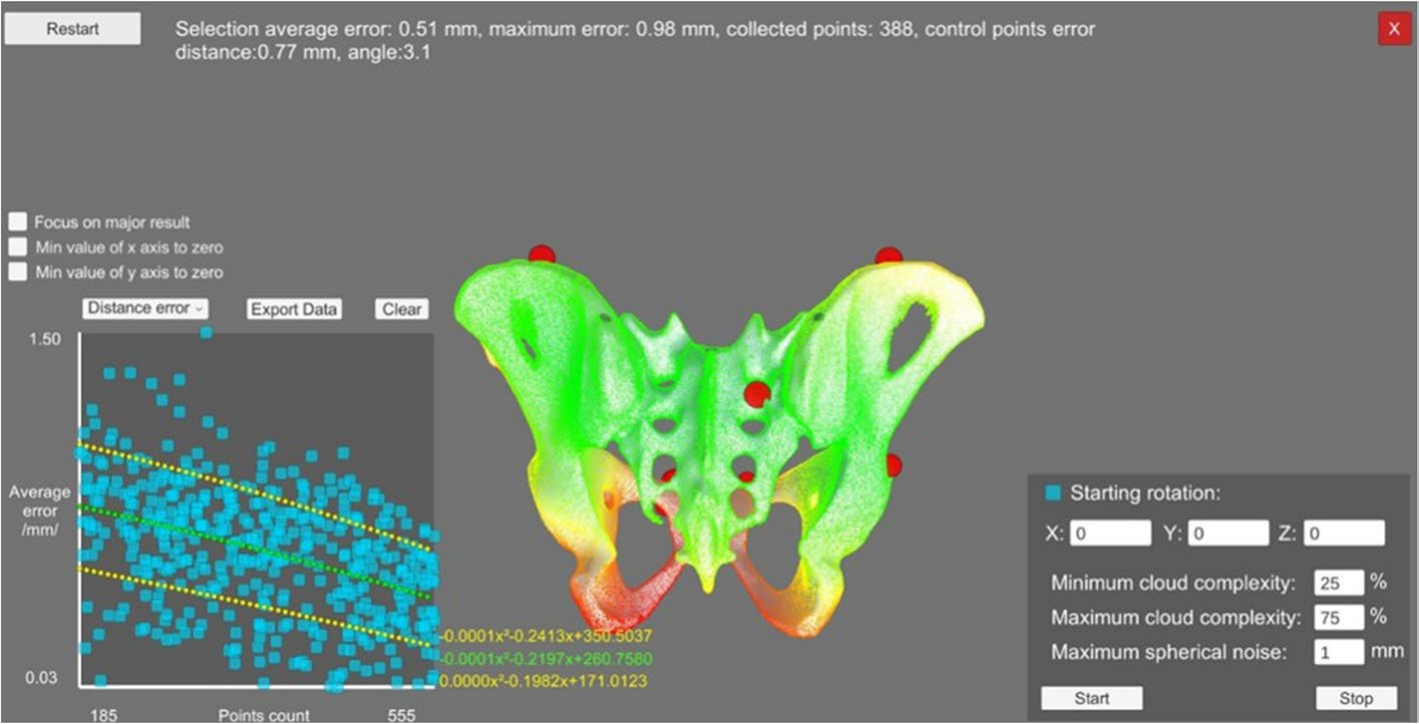
Quality of registration using an optimized surface matching algorithm; result of preoperative simulation of registration accuracy

An optimized surface registration algorithm was used to register the patient’s anatomy, with an ArUco marker attached to the left anterior iliac crest. This method was designed for later use in actual interventions and will be discussed in a separate manuscript. The average error of the registration procedure was estimated at 0.51 ± 0.47 mm (Fig. 4). Surgical Tools were calibrated using a metal calibration frame [16].

**Fig. 5:**
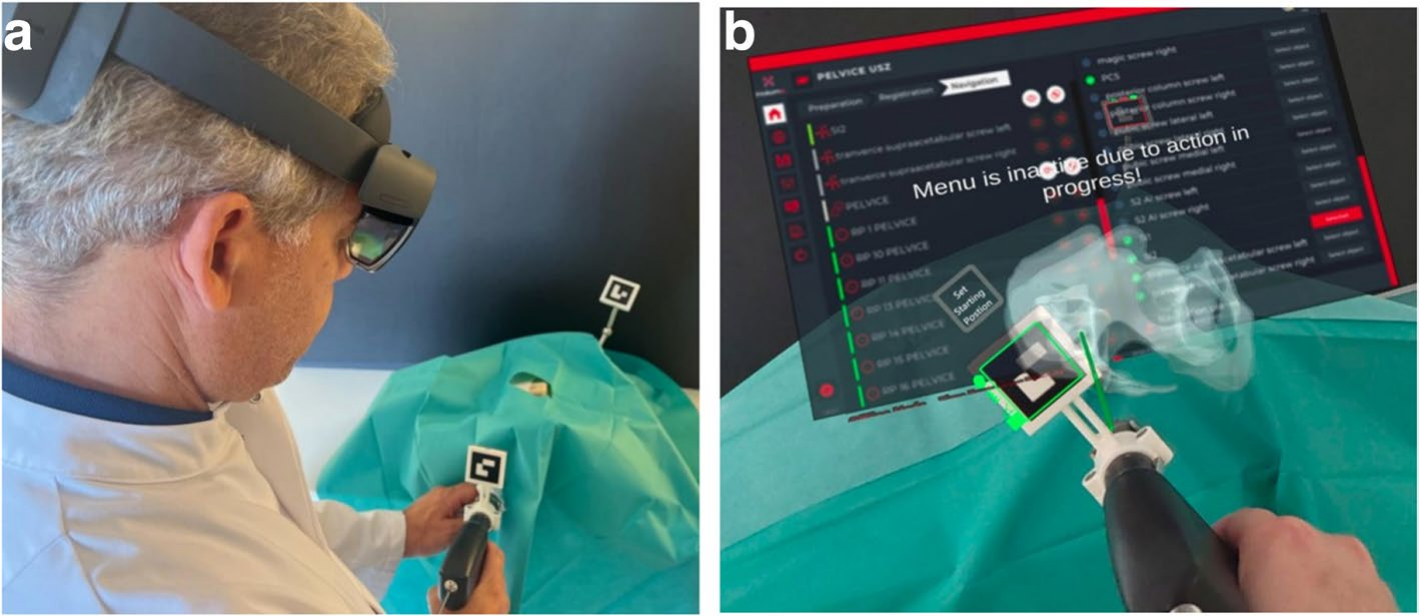
**a**) Experimental setup and **b**) surgeon’s view while drilling

The HoloLens 2-based navigation system allowed the surgeon to track the instruments in real-time using virtual guides (Fig. 5).

The system allows for choosing different visualization modes. The visualization of virtual objects that were defined before, e.g., cortical and cancellous bone and surgical instruments, can thus be turned on/off. This modular structure of the software allows fast adaption to different surgical tasks and makes HoloMA an ideal tool for the development of AR-based procedures [14].

Figure 5b demonstrates the so-called “transparency mode,” which allows intuitive control of surgical instruments in a given 3D anatomy. Note that no extra multi-planar visualization is needed [10].

Both a drilling machine (Colibri, Synthes, Switzerland) and a conventional drill sleeve guide with a diameter of 2,7 mm (7,3 mm cannulated screw system (DePuy Synthes, Switzerland) were converted into an AR-trackable instrument by mounting an adaptor with an ArUco marker (Fig. 2). The navigation was performed based on the drilling machine’s or the drill sleeve’s position and orientation, which was acquired in real-time with the HoloLens 2 camera and ArUco marker detection (Fig. 5b).

Deviation from the entry point and deviation from the planned trajectory were visualized by red lines, which, in case of correct orientation and entry point placement, were turned into green points [17]. Drilling was performed when the first green point was lying on the entry point and the second one on the tip of the actual instrument trajectory. Furthermore, a deviation of the drill from the planned trajectory during the drilling process was visualized by the red lines. A cylinder with the length and diameter of the instrument or diameter of the target directory represented any drill bit, tool, or K-wire.

### Data analysis

After AR-navigated drilling of the channels with 3.2 mm K-wires, they were filled with 2.0 mm pencil lead (Faber-Castell 2 mm HB) and sealed with cyanoacrylate [18]. After that, CT scans of the four pelvic phantoms were obtained using the identical scan protocol.

Data evaluation was performed using 3D image-based engineering software (Mimics Innovation Suite 23.0, Materialise NV, Leuven, Belgium). In reverse engineering, both planned and realized trajectories were visualized in the 3D software, and accuracy measurements were performed by two independent observers (VR, SMH).

**Fig. 6:**
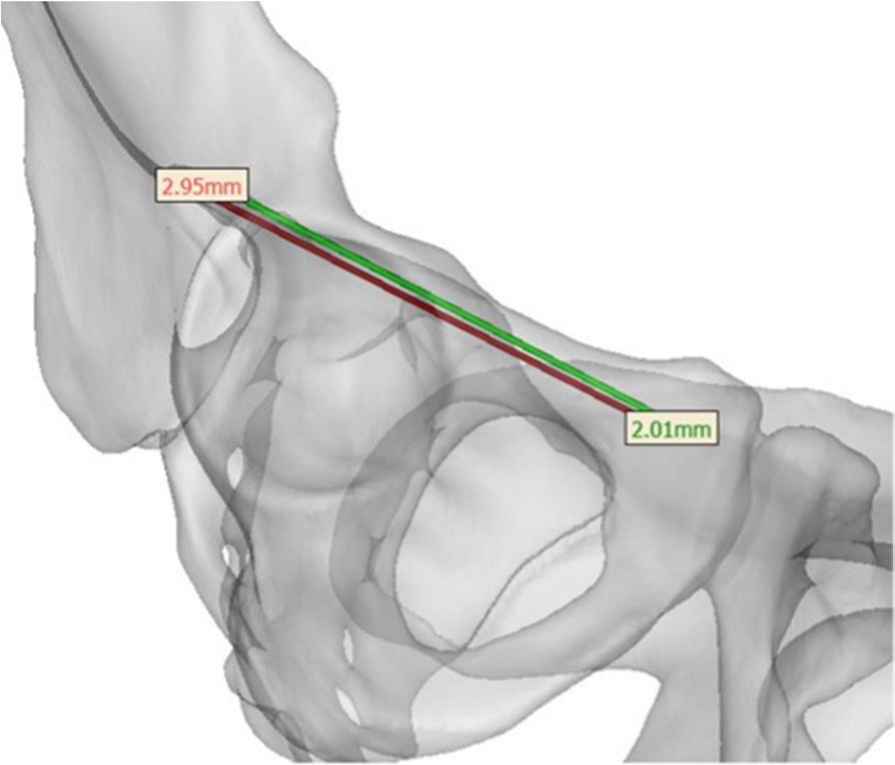
Quantification of deviations using Mimics: the green line represents the planned drilling, the red line the realized drilling. ∆entry point is indicated in mm in green numbers, ∆exit point in mm in red numbers

**Fig. 7:**
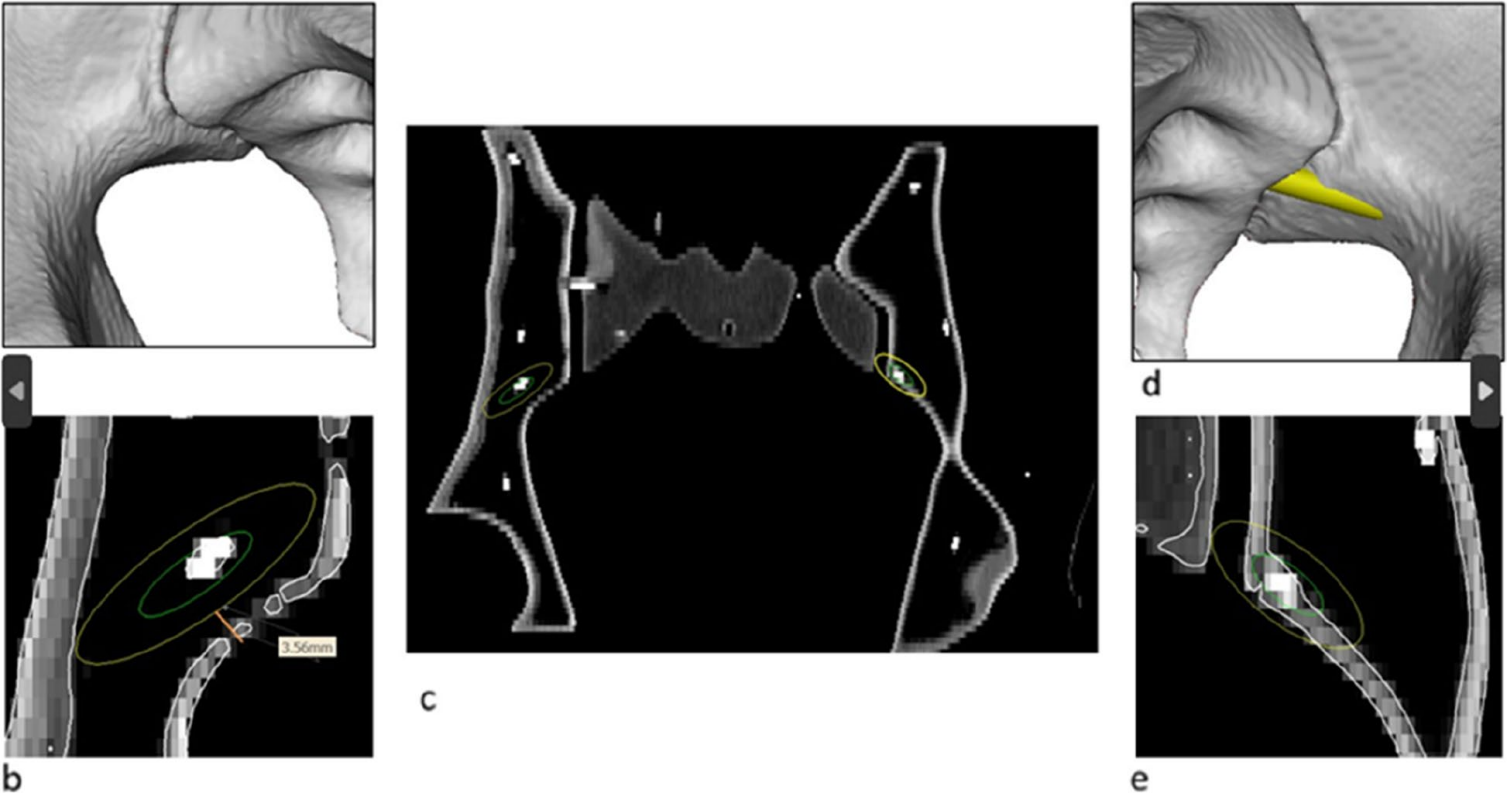
Measurement of the minimal distance to the external cortex in specific cross sections of the bone along the screw trajectory: **a**) right side S2AI-screw, Grade 0, 3D surface visualization; **b**) right side S2AI-screw, Grade 0, multiplanar reconstruction with trajectory and simulated screw diameter 8.5 mm; **c**) both sides in comparison; **d**) left side S2AI-screw, Grade 2, 3D surface visualization; **e**) left side S2AI-screw, Grade 2, multiplanar reconstruction

Postinterventional measurements included translational and rotational deviations of realized trajectories, including entry-point deviation (∆EN point planned/realized), exit-point deviation (∆EX point planned/realized) (Fig. 6), deviation angle (∆angle planned/realized), the screw length (∆realized/planned) and the least perpendicular distance from the outer diameter of each simulated screw drill path to the bony cortex (Fig. 7b).

**Fig. 8:**
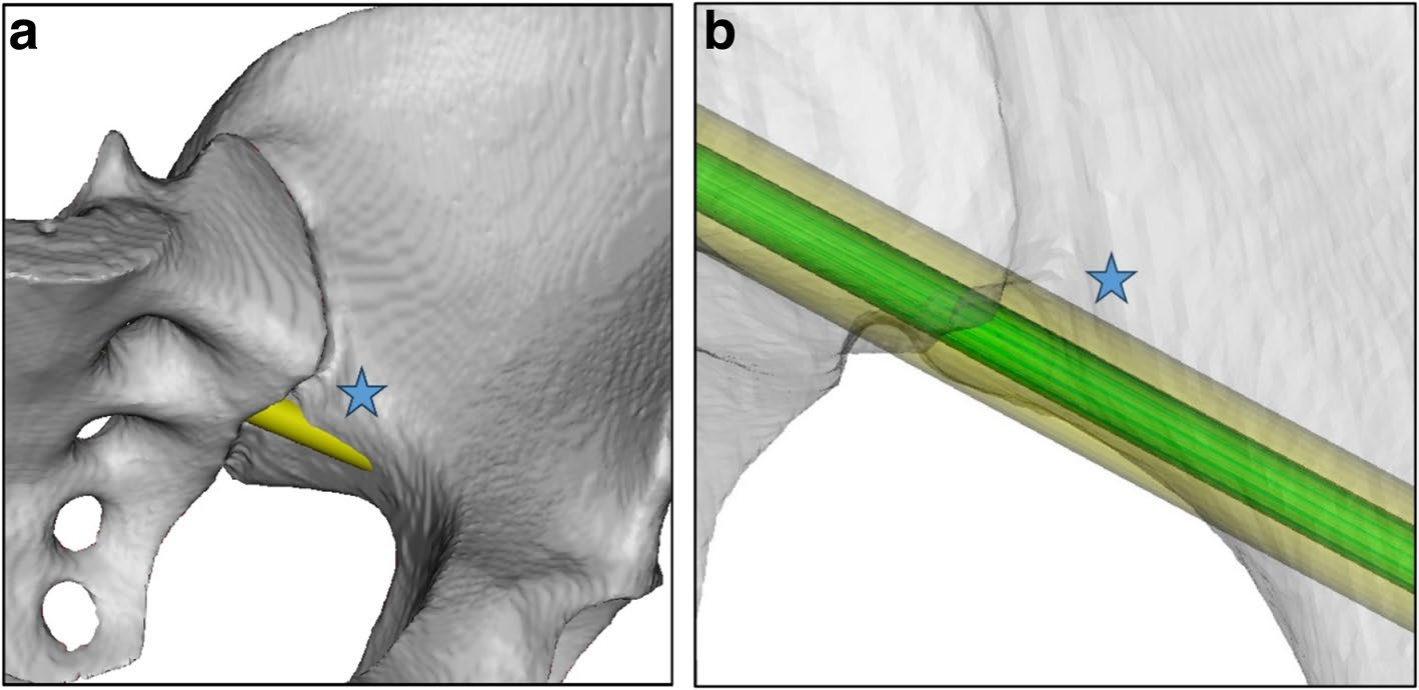
Example of PSPS Grade 2: **a**) left side S2AI-screw, Grade 2, 3D surface visualization; **b**) left side S2AI-screw, Grade 2, 3D surface visualization with trajectory in green and simulated screw diameter in yellow color

This distance to the cortex allowed a CT-based virtual calculation of the perforation risk, using our “pelvic screw perforation score” (PSPS) and taking into account different virtual screw diameters (3.5 mm; 6.5 mm, 7.3 mm, and 8.5 mm) according to the respective anatomical screw position Fig. 8.

The “pelvic screw perforation score (PSPS)” was developed by adaptation of the Gertzbein and Robbins classification of spinal pedicle screw placement and defined as follows (Table 2) [19, 20]:

**Table 2:** Pelvic Screw Perforation Score (PSPS)

**Grade 0:**	The screw is entirely inside the cancellous bone without contact with the internal surface of the cortical lamina.
**Grade 1:**	The screw penetrates the internal cortical lamina but not its external surface.
**Grade 2:**	The screw partially penetrates the external cortical lamina, with less than **3 mm**; furthermore, the tip of the screw ends within the cancellous bone.
**Grade 3:**	The screw penetrates the external cortical lamina in a range between **3 and 6 mm,** with the tip of the screw ending within the soft tissues.
**Grade 4:**	The screw penetrates the external cortical lamina with more than **6 mm.**

The IBM software SPSS Statistics (Version 28.0.1.1) was used for statistical analysis. The t-test for unpaired samples was used to prove the independence of translational, longitudinal, and rotational deviation in the right and left drill pathways. Mean values, standard deviations, confidence intervals, and correlations were calculated using Microsoft Excel (Microsoft Office Professional Plus 2016).

## Results

In this experimental setup, AR-navigated percutaneous drilling of 12 screw pathways per hemipelvis in 8 hemipelves was performed.

Outcome parameters are overall success rate, accuracy in lateral translation, rotational deviation and deviation in longitudinal translation, screw length, and the Pelvic Screw Perforation Score, an adaption made to this anatomic region from the Gertzbein and Robbins classification [19, 20].

Out of 96 possible entry points, 90 were identified correctly, resulting in a success rate of 93.8%, while 83 out of 96 pathways could be finished successfully (86,5%).

The t-test for unpaired samples was used to prove the independence of translational, longitudinal, and rotational deviation in the right and left drill pathways. The analysis yielded a t-value of 0.4513. The corresponding *p*-value was 0.6605, which was significantly above the value of 0.05 or 5%, respectively. The null hypothesis could be kept. Both sides, the left and the right side, behaved identically, and drill pathways could be examined combined.

Accuracy was measured as the deviation of entry point (accuracy representing lateral translation deviation), deviation of angle (rotational deviation), and exit point deviation (rotational deviation plus deviation in longitudinal translation). The overall mean value of translational deviation was 3.99 ± 1.04 mm, the overall mean value of the deviation of the angle was 4.3 ± 1.8°, the overall mean value of exit point deviation was 4.8 ± 0.8 mm.

The overall mean values and the overall standard deviation of all 12 drill pathways were taken.

For specific ∆EN point values for the different drill pathways, see Table 3.

**Table 3:** Specific ∆EN point values for the 12 different drill pathways

Screw position	Drill Pathway#	mean ∆EN	STD	95% CI
Iliac crest anterior screw	1	4.19	1.76	2.71–5.66
Iliac crest posterior screw	2	5.82	2.59	3.66–7.98
Pubic medial screw	3	3.09	1.33	1.98–4.20
Pubic lateral screw	4	2.9	1.09	1.99–3.80
Iliosacral screw S1	5	3.86	1.93	2.24–5.47
Iliosacral screw S2	6	5.02	2.47	2.95–7.08
Transverse supraacetabular screw	7	5.45	1.95	3.82–7.09
Supraacetabular screw	8	4	1.68	2.60–5.41
Anterior column screw	9	3.68	1.62	2.32–5.03
Posterior column screw	10	2.3	1.16	1.32–3.27
Quadrilateral plate („magic“) screw	11	4.1	2.08	2.37–5.84
S2-alar-iliac screw	12	3.45	1.53	2.18–4.73

**Fig. 9:**
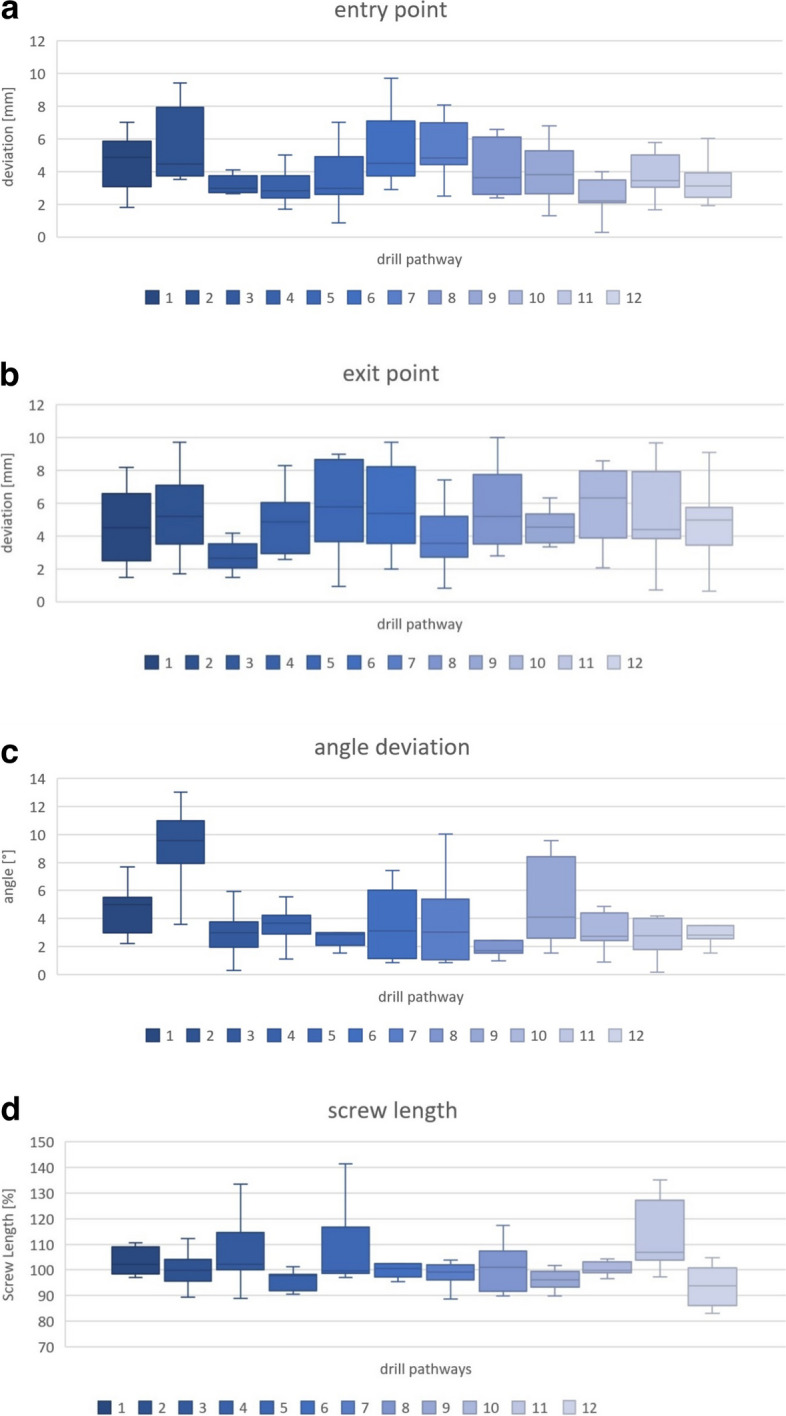
Results for **a**) ∆EN, **b**) ∆EX, **c**) ∆angle, **d**) ∆screw length

Exit point deviation was a combined error of entry point deviation (lateral translation), angle deviation (rotational), and depth navigation (longitudinal) and resulted in a variation of screw length. The mean screw length achieved versus planned was 103 ± 8% Fig. 9.

Out of 96 planned screw pathways, 88.7% were realized in accordance with PSPS Grade 0 and 1 and 100% in accordance with Grade 0–2, which was considered satisfactory operation results. Grade 0–1 was considered without theoretical risks for neurological symptoms or vascular damage independent of the screw type, while Grade 2 requires particular attention as discussed later [19, 20].

Correlations between the different parameters were tested as being weak. This indicates that the test persons (3 surgeons) showed no significant correlation. Drill path accuracy was independent between different test persons and independent from the side.

## Discussion

This study was designed to demonstrate the feasibility of minimal-invasive drilling of screw pathways in the pelvis using an AR surgical navigation system consisting of an off-the-shelf optical-see-through HMD, a surgical visualization software client that runs on the HMD, and a standard surgical toolset for this type of procedure attached to tracking markers (ArUco) [14, 15].

In the classification of health technology assessment (HTA), the presented work assessed the therapeutic reliability and interference of the material in a simulated clinical scenario. The motivation for the assessment can be described as the development of surgical techniques towards minimally invasive procedures and the need for precise image guidance on the one hand and the availability of novel HMDs on the other hand. The system to be assessed and the surgical context and intended use of the system have been described in detail [21, 22].

While the system proved to be robust, with an overall success rate for entry-point navigation of 93.8%, the drop-out rate of 6.2% has to be explained. The few drop-outs resulted from line-of-sight problems and workflow disruptions caused by the oversensitive user interface (UI) of HoloLens 2 (known as wrist tap mode), which could be corrected by software modifications (HoloMA version 1.5.0) [23]. Similar problems have been described by other teams to a comparable extent [24].

Notably, the entry points of screw trajectories that showed larger variation in several parameters (e.g., drill pathways #2 “iliac crest posterior screw,” #6 “iliosacral screw S2,” #7 “transverse supraacetabular screw”) are located in the lateral or dorsal aspect of the pelvis. This 360° orientation of drill pathways in the pelvis of our test setup based on just one registration is technically very challenging compared with a typical clinical setting where the workflow is either anterior/posterior, posterior/anterior, or side to side with the navigation system registered ideally to the respective drilling task. Another reason is the specific slope of the plastic phantoms´ surface in these regions, generating a higher probability of slipping at the entry point. Moreover, slipping can also occur due to missing soft tissue covering in the experimental setting.

Modifications in the registration process towards an optimized point cloud registration need to be discussed separately. In our setting, an optimized surface-matching routine was used (Fig. 4), which will allow transfer to the OR in the future [16].

To be able to compare navigation accuracy, the technical limitations of the system have to be known. In a recent publication, technical specifications of surgical tool tracking with off-the-shelf AR HMDs have been described in detail [24]. Tool tracking accuracy of spherical retro-reflective markers showed superiority compared to ArUco markers for lateral translation, while ArUco markers performed relatively stable in rotation. This limitation has to be accepted in our setup. For translational as well as rotational deviation, our results in the specific clinical setup of pelvic trauma surgery are comparable to other groups using visual guidance for K-wire placement [24]. The use of spherical reflective markers might be beneficial and should be considered.

While results in translational and rotational accuracy can be well explained with the known technical properties of ArUco marker tracking, the screw length achieved is a resulting parameter. The achievable screw length according to intraosseous position has clinical relevance, mainly with respect to the stability of the construct. Too short screws may result in early failure and loosening, e.g., in the case of symphysis plates. Too long screws may lead to penetration of the far cortex and potentially damage neuro-vascular structures. Anchoring of implants is most important in poor bone quality and a significant issue in the development of surgical procedures. Here AR-navigated screws achieved this goal above expectation [25–27].

A clinically valuable perforation score for pelvic trauma surgery was described here as the “pelvic screw perforation score (PSPS).” This tool allows the classification of drilled pathways. The perforation risk depends on the anatomic features of the respective pelvic region and the desired diameter of the screw to be placed in that anatomy. Together with the given system accuracy, it can now be determined whether the technique is suitable for the specific surgical task [25–29].

**Fig. 10:**
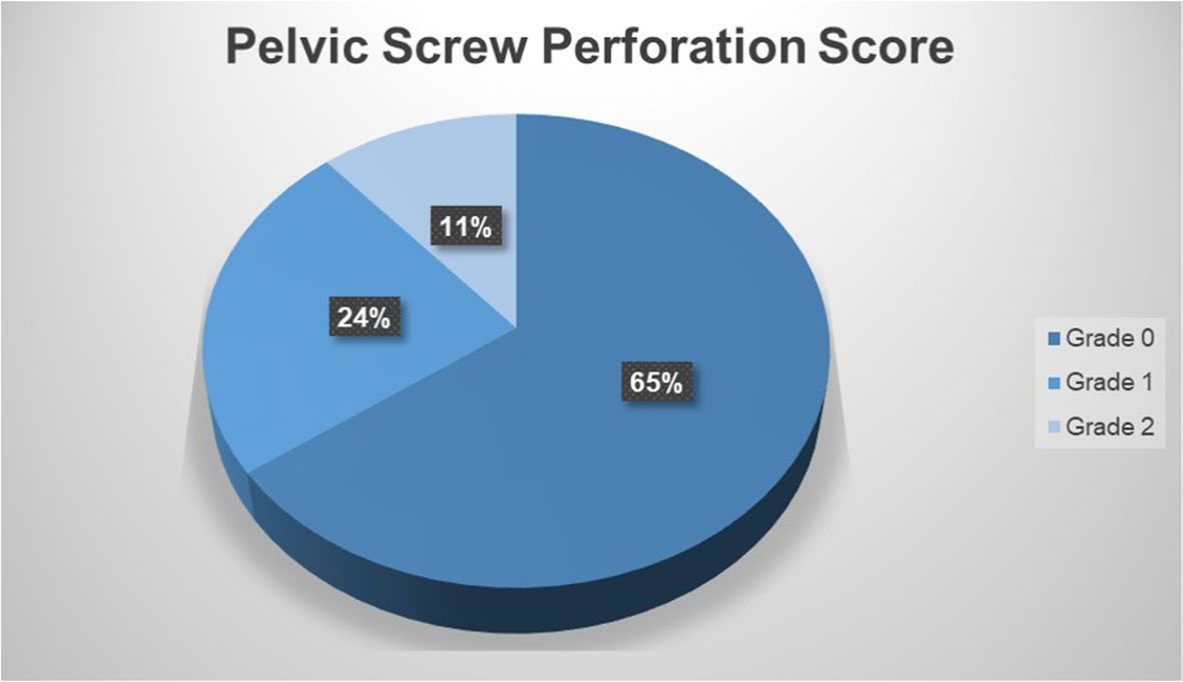
Grading of screw trajectories according to Pelvic Screw Perforation Score

For our setup and the off-the-shelf HMD with HoloMA software, the results of the screw perforation score (Fig. 10) were mostly within the clinically safe range. Theoretically, no neurological or vascular damage has to be expected with screw positions in grade 0–1, which could be achieved within 89% [19, 20].

Concerning perforation grade 2, the assessment of theoretical risk for critical structures largely depends on the screw trajectory. While for some screws, a cortical perforation of less than 3 mm will not result in neurovascular or organ damage, some screws might cause injury of nerves, e.g. perforation of the sacral ala by iliosacral screw S1 hitting the L5 nerve root or perforation of the S1 foramen with injury to S1 nerve root, or vessels, e.g. injury of corona mortis by perforation of the anterior column screw. Therefore, in the clinical situation, the process of registration must continually be optimized to the respective screw pathway drilled. In this experimental setup, surface registration was done only once per pelvis at the beginning of the experiment and used for all 24 screws, with the reference marker in the left anterior iliac spine.

These results suggest a substantial equivalence in performance with other computer-assisted methods based on intraoperative 3D data and they are superior to conventional techniques and 2D navigation [7–9, 30].

Published clinical results from a CT study show high variation in entry point positioning due to 14-38 mm variation in the horizontal plane and 9–15.9 mm in the vertical plane for the entry point in AC column screws [27]. The comparison of translational screw deviation between conventional technique 5.1 ± 3.0 mm, 2D-fluoroscopy based navigation 5.5 ± 3.0 mm, and 3D-fluoroscopy based navigation 4.7 ± 2.5 mm has also been examined before [9]. Malpositioning rates were described as 5–24% with the conventional technique, 2–14% with 2D-fluoro navigation, and 0–5% with CT navigation, respectively [8].

Our results with translational screw deviation for the entry point of 3.99 ± 1.04 mm and 88.7% of screws following PSPS Grade 0 and 1 and 100% following Grade 0–2 prove the strong performance of our concept.

AR-based navigation can build a bridge between pre-and intraoperative 3D scans at discrete points in time, thus avoiding additional radiation exposure for the surgeon and the OR staff. As our study pioneered this technique, time issues were not in focus, but time expenditure seems comparable to conventional navigation systems. There is still a need for matching and tool calibration first. In our experimental setup, we also had a preoperative planning step to measure deviations. The user interface can easily be controlled entirely within the sterile field, and our favorite visualization mode with semitransparent bone visualization enables intuitive navigation with conventional tools and a non-altered workflow.

A standard surgical toolset has been used, and the attached markers were designed to be sterilizable. Tools were implemented and calibrated in a fast software routine using a calibration frame directly before surgical navigation. This method allows for maximum flexibility and enables easy adaption of the system to a variety of surgical tasks. In that way, the software has proven to be a powerful tool for the development of specific AR procedures. In our study, a complete set of 24 pathways could be visualized simultaneously, and each drill pathway could also be addressed individually to simplify workflow and support the surgeon’s attention. The most desired visualization mode is 3D navigation in transparency mode, enabling “in-situ”-visualization of a surgical tool trajectory within the 3D anatomy [11, 31].

HoloMA software has a unique multiplayer mode that allows to share one coordinate system and enables cooperation in the virtual space. This specification simplifies not only cooperation in an experimental setup but also facilitates watching of AR procedures. This is an important option that allows us to practice the “shadow surgeon“- concept [32].

A noticeable side effect of 3D navigation is its benefit for teaching and education. Visualizing complex 3D anatomy in the transparency mode helps to generate a good perception of the specific anatomical situation and screw pathways in particular. Training setups with commercially available phantoms can easily be accomplished. The modular structure of the software allows for easy modification and adjustment to different surgical situations and instruments.

Our setup respects many aspects of possible intraoperative use, like workflow, specific surgical instruments, calibration routine and sterilization of components and markers.

The user interface allows sterile control of the complete workflow.

Although our system is nearly ready for clinical use there is still a need for regulation and compliance with oversight bodies.

## Conclusion

The AR navigation system proved feasibility for surgical navigation in minimal-invasive pelvic surgery in 12 anatomically demanding pathways. It showed comparative performance with other computer-aided surgical navigation solutions, with specific benefits like true 3D navigation in the transparency mode and potentially lower ionizing radiation exposure during the procedure.

The system design setup respects OR conditions; however, it needs further improvement and must still undergo regulatory body approval. Future endeavors include intraoperative registration and optimized tool tracking.

## Data Availability

On request from the corresponding author.
